# Isolation and Characterization of Porcine Sapelovirus from the PDCoV-Positive Sample and Its Molecular Epidemiology in Henan Province, China

**DOI:** 10.1155/2023/9943040

**Published:** 2023-04-25

**Authors:** Yunfei Zhang, Qianqian Li, Lulu Si, Junlong Gao, Jin Yuan, Lu Xia, Hui Hu

**Affiliations:** ^1^The College of Veterinary Medicine, Henan Agricultural University, Zhengzhou, Henan 450002, China; ^2^Key Laboratory for Animal-Derived Food Safety of Henan Province, Zhengzhou, Henan 450002, China

## Abstract

Porcine sapelovirus (PSV) is an emerging swine enteric virus that can cause various disorders including acute diarrhea, respiratory distress, reproductive failure, and polioencephalomyelitis in pigs. In this study, we isolated a PSV strain HNHB-01 from a clinical porcine deltacoronavirus- (PDCoV-) positive intestinal content of a diarrheic piglet. PSV was first identified using the small RNA deep sequencing and assembly, and further identified by the electron microscopic observation and the immunofluorescence assay. Subsequently, this virus was serially passaged in swine testis (ST) cells, and the complete genomics of PSV HNHB-01 passage 5 (P5), P30, P60, and P100 were sequenced and analyzed. 9 nucleotide mutations and 7 amino acid changes occurred in the PSV HNHB-01 P100 strain when compared with the PSV HNHB-01 P5. Pathogenicity investigation showed that orally inoculation of PSV HNHB-01 P30 could cause obvious clinical symptoms and had broad tissue tropism in 5-day-old piglets. Epidemiological investigation revealed that PSV infections and the coinfections of diarrhea coronaviruses were highly prevalent in swine herds. The complete genomes of 8 representative PSV epidemic strains were sequenced and analyzed. Phylogenetic analysis revealed that the PSV epidemic strains were closely related to other PSV reference strains that located in the Chinese clade. Furthermore, recombination analysis revealed that the recombination events were occurred in downstream of the 2C region in our sequenced PSV HNNY-02/CHN/2018 strain. Our results provided theoretical basis for future research studies of the pathogenic mechanism, evolutionary characteristics, and the development of vaccines against PSV.

## 1. Introduction

Porcine sapelovirus (PSV) belongs to the genus *Sapelovirus* of family *Picornaviridae* [1]. Currently, the *Sapelovirus* genus mainly includes three species, Sapelovirus A formerly known as porcine sapelovirus, Sapelovirus B named as simian sapelovirus, and Avian sapelovirus represented by duck picornavirus TW90A [1, 2]. In addition, several unclassified sapeloviruses have been reported, including bat, California sea lion, marmot, mouse, and WUHARV sapeloviruses [3]. PSV is a single-stranded, nonenveloped RNA virus with the genome length of 7.5∼8.3 kB. The genomic organization is similar to other picornaviruses, including a 5′ untranslated region (UTR), a large open reading frame (ORF), a 3′ UTR, and a poly (A) tail [4]. The ORF consists of a single polyprotein that is subsequently processed into four structural proteins (VP4, VP2, VP3, and VP1), seven functional proteins (2A, 2B, 2C, 3A, 3B, 3C, and 3D), and a leader protein (L) [5–8].

PSV can cause acute diarrhea, pneumonia, polioencephalomyelitis, and reproductive disorders in pigs. Several reports indicated PSV infections can cause asymptomatic and symptomatic diseases in both field and experimental pigs [9]. PSV consist only one serotype, and domestic pigs and wild boars are the only species known to be naturally susceptible to PSV [4]. Previous reports have shown that PSV can cause severe watery diarrhea, accompanied by polioencephalomyelitis syndrome (ataxia and leg paralysis) in piglets [4, 6, 10]. PSV infections in pigs have been reported in several countries around the world, including European countries, Japan, China, Korea, Brazil, India, and United States [4–6, 9, 11]. The prevalence rates of PSV among swine herds in India and Hungary are 7.1% and 71.0%, respectively [12, 13]. To date, little is known about the molecular epidemiology of PSV in China, especially in Henan, a major pig-raising province in China.

Coinfection of PSV with other swine enteric viral pathogens is frequently reported, the coinfection of PSV and porcine epidemic diarrhea virus (PEDV) had been reported and the prevalence rate was 20.3% in diarrhea samples [8], and the coinfections of PSV with porcine teschovirus (PTV) and porcine enterovirus (PEV) have been reported in asymptomatic or in association with symptoms in pigs [11, 14], which indicated that PSV is wide-spreading in pig populations. However, no treatments or vaccines are available for PSV currently.

The isolation of PSV strains was useful for studies of pathogenesis, and for the development of detection methods and vaccines. PSVs have been isolated and propagated in some primary porcine cells, and a number of continuous cell lines. Apart from swine original cell lines, PSVs can also replicate in baby hamster kidney (BHK-21) and human embryonic kidney (293 T) cell lines [6, 8, 15, 16]. PSV uses *α*2,3-linked sialic acid on GD1a ganglioside as a receptor to enter the LLC porcine kidney (LLC-PK) cells [9]. However, PSV can enter intestinal epithelial cell line (IPEC-J2) cells by caveolae-mediated endocytosis, and the host syndecan-1 protein can promote PSV VP1 synthesis and virus replication [17].

In the present study, a Chinese PSV strain HNHB-01 was isolated from the piglet's intestinal contents collected from diarrheic piglets with coinfection of PDCoV in Henan province, China. The characterization of the PSV HNHB-01 strain was determined and the complete genomes of the cell culture-adapted PSV passage 5, 30, 60, and 100 were analyzed, and then, the pathogenicity of PSV HNHB-01 P30 strain was investigated in 5-day-old piglets. Moreover, the prevalence of PSV in Henan Province was investigated. At last, the eight representative PSV strains were sequenced and analyzed, and the recombination was detected using recombination detection program version 4 (RDP4) and Simplot. This study may provide novel information for the investigation of the swine enteric viruses' coinfections and the potential targets for the development of antiviral agents and vaccines for PSV infections.

## 2. Materials and Methods

### 2.1. Cells

The ST and LLC-PK cell lines were purchased from the Institute of China Veterinary Medicine Inspection, and used to isolate PSV in the current study. The growth medium for LLC-PK cells was minimum essential medium (MEM) supplemented with 5% heat-inactivated fetal bovine serum (FBS, Gibco, USA), 1% of antibiotic-antimycotic, *N*-2-hydroxyethylpiperazine-*N*-2-ethane sulfonic acid (HEPES), and MEM nonessential amino acids solution (NEAA) (Gibco, USA). The ST cells was cultured in Dulbecco's modified eagle medium (DMEM, Gibco, USA) supplemented with 5% FBS (Gibco, USA) and 1% antibiotic-antimycotic (Solarbio, China).

### 2.2. Virus Isolation and Propagation

A total of 26 PDCoV-positive intestinal content samples were collected from commercial pig farms in Henan Province, China, and identified by our laboratory using PDCoV-specific RT-PCR [18], and these PDCoV-positive samples were also negative for PEDV, transmissible gastroenteritis virus (TGEV), porcine circovirus type 2 (PCV-2), and porcine reproductive and respiratory syndrome virus (PRRSV) by viral-specific PCR assays [19]. The PDCoV-positive samples were diluted 5-fold with MEM including 1% antibiotic-antimycotic. The mixture was centrifuged at 1,847*g* at 4°C for 20 min. The supernatants were filtered through 0.22 *μ*m filters and used as inoculum for virus isolation on cells. When the cell monolayers were grown to 90% confluence in 6-well cell-culture plates around 24 h, cells were washed twice with D-Hanks. The filtered samples were added to the 6-well plates. Plates were incubated for 50 min at 37°C in 5% CO_2_ incubator. After twice washing with D-Hanks, DMEM containing 1% antibiotic-antimycotic and 1% pancreatin (Sigma-Aldrich) for the ST cell, and MEM including 1% antibiotic-antimycotic, HEPES, NEAA, and 5 *μ*g/mL trypsin (Sigma-Aldrich) for LLC-PK cell were added to the cells, respectively. The plates were further incubated at 37°C in 5% CO_2_ and monitored for cytopathic effect (CPE). When the CPE reached to 80–90%, the plates were frozen at −80°C and thawed twice. The cultures were harvested and used as seed virus for further passage on ST and LLC-PK cells [20, 21].

### 2.3. RNA Extraction and Deep Small RNA Sequencing and Assembly

The total RNA was extracted from the plaque-purified viruses by RNA plus Reagent (TaKaRa, China) following the manufacturer's instructions. The extracted RNA was subjected to next generation sequencing on an Illumina MiSeq platform (Jinweizhi Biological Technology Co. Ltd.). Viral RNA was quantified and qualified by Agilent 2100 Bioanalyzer (Agilent Technologies, USA), NanoDrop (Thermo Fisher Scientific Inc.), and 1% (w/v) agrose gel. 2 *μ*g total RNA with RIN value above 7.5 was used for following library preparation. Next generation sequencing library preparations were constructed according to the manufacturer's protocol (NEBNext® Multiplex Small RNA library Prep Set for Illumina®).

3′ SR Adaptor for Illumina was ligated to the small RNA using 3′ Ligation Enzyme. 5′ SR Adaptor for Illumina was ligated to the small RNA using 5′ Ligation Enzyme and first strand cDNA was synthesized using ProtoScript II Reverse Transcriptase. Each sample was then amplified by PCR for 12 cycles and the PCR products of ∼140 bp were recovered and cleaned up using PAGE, validated using an Agilent 2100 Bioanalyzer, and quantified by Qubit 2.0 Fluorometer. Then, libraries with different indexes were multiplexed and loaded on an Illumina HiSeq instrument. Sequencing was carried out using a 1 × 50 single-end (SE)/2 × 150 paired-end (PE) configuration. Image analysis and base calling were conducted by the HiSeq Control Software (HCS) + OLB + GAPipeline-1.6 (Illumina) on the HiSeq instrument.

### 2.4. RT-PCR and qRT-PCR for PSV Detection

To determine whether the virus detected by small RNA deep sequencing and assembly in the cell culture supernatant was PSV, the PSV-specific primers targeting PSV 5′ UTR (PSV-F5′-GACTTGACGAGCGTCTCTTT-3′ and PSV-R5′-CACGGGCTCTCTGTTTCTT-3′) were used for the RT-PCR. The RT-PCR program was as follows: 95°C for 5 min; 35 cycles of 95°C for 30 s, 56°C for 30 s, 72°C 90 s, and 72°C for 5 min, 4°C for 15 s.

qRT-PCR was carried out using the specific primer targeting PSV 5′ UTR (PSV-F5′-CGACTGGTTACAGGAGAGTAGA-3′ and PSV-R5′-TAAGGTACACACGGGCTCT-3′). The qRT-PCR program was: 95°C for 30 s; 40 cycles of 95°C for 15 s, 58°C for 30 s, and 95°C for 15 s, 60°C for 60 s, 95°C for 15 s.

### 2.5. Electron Microscopy (EM) Observation

The virion particles in the PSV-infected LLC-PK cell culture were observed using electron microscopy. Briefly, the PSV-infected cell culture was frozen and thawed three times and centrifuged at 5,000*g* at 4°C for 20 min. Then, the supernatants were collected and filtered through 0.22 *μ*m filters. PEG 6,000 (8%, w/v) and NaCl (0.5 M) were added to the medium, and stirred for 3 h at 4°C by the magnetic stirrer with a beaker in the medium. The mixed liquor was subjected to centrifuge at 30,000*g* for 4 h at 4°C, and the precipitate was resuspended in phosphate buffer saline (PBS). Then, the viral particles were negatively stained with 2% phosphotungstic acid and examined with a transmission EM (Hitachi H-7000FA, Japan) [20, 22].

### 2.6. Virus Purification by a Plaque Assay

The plaque assay for PSV was established according to our previous reported method [22]. ST cells in 6-well plate were grown to 100% around 24 h. After washing twice with D-Hanks, DMEM (2 mL/well) was added to cells, and the plates were incubated for 1 h at 37°C in 5% CO_2._ The diluted viruses were added to each well (400 *μ*l/well). After 1 h incubation at 37°C in 5% CO_2_, cells were washed with D-Hanks. 2 mL of agarose-MEM overlay solution per well (equal volume of 2% (w/v) seaPlaque agarose (Lonza, Rockland) and 2 × MEM (Gibco, USA) containing 1% antibiotic-antimycotic and 2% pancreatin). According to the plaque development, the plates were stained with 0.01% neutral red (Sigma-Aldrich) for 30–60 min at 37°C in 5% CO_2_. The plaques were picked by using tips and incorporated into 0.5 mL maintenance medium and stored at −80°C [21].

### 2.7. Immunofluorescence Assay (IFA)

PSV was used to infect ST cells in 12-well plate. When CPE was about 20–30%, cells were washed with D-Hanks and fixed with 100% ethanol at 4°C for 4 h or overnight. After washing twice with D-Hanks, cells were permeabilized in 0.05% Triton X-100 at room temperature for 15 min. Subsequently, the 5% (w/v) bovine serum albumin (BSA) in PBS was added to cells and incubated at 37°C for 2 h. Then, cells were incubated with anti-PSV VP1 specific monoclonal antibody (prepared in our lab, 1 : 100 dilution) overnight at 4°C, the plates were washed 5 times with PBS containing 0.05% Tween-20 (PBST). After added fluorescein-labeled antimouse IgG antibodies (H + L) (1 : 100 dilution) at 37°C for 1 h, the plates were washed 5 times with PBST. Cellular nuclei were stained with DAPI (Solarbio, China) for 10 min at the room temperature. Then, the plates were washed 5 times with PBST. Cell staining was examined using a fluorescence microscope (Olympus, Germany) [21].

### 2.8. One-Step Growth Curve of PSV HNHB-01 Strain on ST Cells

When the ST cells in 6-well plate were reached 90–100% confluent, the plates were washed three times with D-Hanks. Cells were inoculated with PSV HNHB-01 strain at multiplicity of infection (MOI) of 0.01. After adsorption for 1 h in a CO_2_ incubator at 37°C, cells were washed three times with D-Hanks and 2 mL of maintenance medium (DMEM containing 1% pancreatin) was added to the cells. Then, the cells and supernatants were harvested together at 6, 12, 24, 36, 48, 60, and 72 h. Subsequently, virus RNA and infectious-virus titers were determined by qRT-PCR and TCID_50_ assays, respectively [21, 22].

### 2.9. Animal Experiments

PSV infection experiments in pigs described herein were performed. Six 5-day-old piglets were purchased from a commercial pig farm. No obvious clinical signs or significant temperature changes were observed in these piglets. Viral specific PCR assays were performed to verify the absence of PSV, PEDV, TGEV, PDCoV, PCV-2, and PRRSV [19]. The protocols for animal experiments on live piglets were approved by the Animal Care and Use Committee of Henan Agricultural University (Zhengzhou, China), and the approval number is HNND2020031012.

The 5-day-old piglets were randomly divided into two groups (*n* = 3 for each group). The PSV-inoculated group was orally infected with 10 mL of PSV HNHB-01 P30 (1 × 10^9^ TCID_50_ per piglet), and the uninfected control group was orally inoculated with 10 mL of MEM. All pigs were evaluated daily for clinical signs and body condition. All piglets were necropsied on 3 days postinoculation (dpi), the samples including lung, duodenum, jejunum, ileum, caecum, colon, and rectum were collected for the pathological examination, and the fresh samples including heart, liver, spleen, lung, kidney, brain, duodenum, jejunum, ileum, caecum, colon, rectum, and mesenteric lymph were served as viral distribution detection.

### 2.10. Histopathology and Immunohistochemistry for the Detection of PSV Antigen

Duodenum, jejunum, ileum, colon, caecum, rectum, and lung were examined grossly and histologically and fixed in 10% neutral formalin for 1-2 days at room temperature. Then, these tissues were embedded, sectioned, and stained with Mayer's hematoxylin and eosin (H&E), and the slides were examined by conventional light microscopy [20, 23].

Immunohistochemistry detection of PSV antigens was performed using anti-PSV VP1 specific monoclonal antibody (prepared in our lab, 1 : 100 dilution), as the primary antibody followed by an incubation with horseradish peroxidase- (HRP-)conjugated goat antimouse IgG secondary antibody (Sigma-Aldrich). Reactions were developed with 3,3′-diaminobenzidine (DAB) reagents. Haematoxylin was used for counterstaining. Dehydration and mounting were conducted with neutral gums [24].

### 2.11. Epidemiological Investigation of PSV in Henan Province, China

For the molecular epidemiological survey of PSV, 460 clinical samples including swine faecal swabs and intestinal contents were collected from asymptomatic or diarrhea pigs in Henan Province. These clinical samples were diluted 5-fold with MEM, and centrifuged for 15 min at 4°C with 1,847*g*. The clarified supernatants were used for RNA extraction. All samples were investigated for PSV, TGEV, PDCoV, and PEDV, respectively. PSV detection was performed using RT-PCR screening for the 5′ UTR as described previously. The method used for detecting TGEV, PDCoV, and PEDV were as previously reported [18].

### 2.12. Complete Genomic Sequence and Phylogenetic Analysis of the PSV Strains

The complete genomes of the representative PSV epidemic strains and the ST cell culture-adapted PSV HNHB-01 P5, P30, P60, and P100 strains were sequenced. The primers were designed according to the sequence of the PSV strain USAIA33375-2015 (Table 1). The genes were amplified by using Phanta® Max Super-Fidelity DNA Polymeras (Vazyme, China). PCR products were purified and cloned into pMD18-T vectors (TaKaRa, China) following the manufacturer's protocol. The positive clone of each amplicon was sequenced twice. The complete sequences of the PSV strains were obtained by assembling overlapping contigs followed by trimming off primer sequence, and then, used for sequence alignments and phylogenetic analyses with other PSV reference strains by using the Clustal W program of DNAStar 7.0 green (DNAstar, Madison, WI). Phylogenetic trees were constructed using the neighbor-joining method in MEGA 6.0 software (http://www.megasoftware.net/) with the bootstrap analysis of 1,000 replicates [18].

### 2.13. Recombination Analysis of PSV Strains

In order to detect possible recombination events of the sequenced PSV strains, the RDP4 package, including RDP, BootScan, GENECONV, Maxchi, SiScan, Chimaera, and 3Seq, was used to analyze the potential recombination events [25]. Subsequently, the recombinant parental strains were analyzed with SimPlot version 3.5.1 [26] with a window size of 200 bp and a step size of 20 bp to identify potential recombination events and breakpoints. The final recombination event was determined after a combined study of the RDP and SimPlot results.

### 2.14. Statistical Analyses

Statistical analysis was performed by SPSS version 17.0 software for windows (SPSS, Chicago, IL, USA). Data are expressed as the mean ± SD for at least two independent experiments.

## 3. Results

### 3.1. Isolation and Propagation of PSV in ST and LLC-PK Cells from the PDCoV-Positive Samples

**Figure 1:**
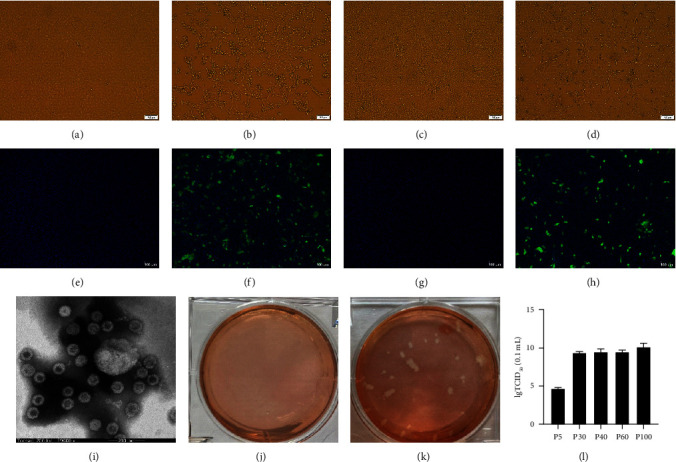
Isolation and identification of PSV HNHB-01 strain. (a–d) The CPE of LLC-PK and ST cells infected with PSV strain HNHB-01. (a) Mock-inoculated with LLC-PK cell culture. (b) PSV- inoculated LLC-PK cells. (c) Mock-inoculated with ST. (d) PSV- inoculated ST cells. (e–h) Detection of PSV isolate HNHB-01 in LLC-PK and ST cells by IF staining using anti-PSV VP1 specific monoclonal antibody. (f) IF staining of PSV HNHB-01-inoculated LLC-PK cells showing large numbers of IF-stained cells. (h) IF staining of PSV HNHB-01-inoculated ST cells showing IF-positive staining. (e and g) IF staining of mock-inoculated LLC-PK and ST cells showing no IF-positive cells. (i) Electron micrographs of PSV HNHB-01-inoculated ST cells. (j and k) Plaques of purified PSV HNHB-01 virions. (l) Virus titers of PSV HNHB-01 on ST cells during serial passage.

LLC-PK and ST cell monolayers were inoculated with 26 of the PDCoV-positive samples. The obvious visible CPEs were observed from one sample which collected from Hebi, Henan Province on ST and LLC-PK cells at 80 h postinfection (hpi). The CPEs were characterized by rounded, clustered, and eventually detached from the cellular monolayers (Figures 1(b) and 1(d)), which were similar to that of PDCoV infection [22]. But this CPE-positive isolate was negative for PDCoV and also negative for other common swine enteric viruses (PEDV, TGEV, swine enteric alphacoronavirus, and porcine rotavirus) when tested by viral-specific RT-PCRs. We further performed next-generation sequencing to screen pathogens in this CPE-positive culture. The result revealed that this isolate was PSV, and no other viral matches were detected.

To confirm the replication of PSV on LLC-PK and ST cells, IFA was conducted using PSV VP1 specific monoclonal antibody (prepared in our laboratory) [27]. The results indicated that the infectious-cells showed large numbers of IF-stained cells (Figures 1(f) and 1(h)). The viral particles in the supernatant from the infected cells were also examined by EM. As shown in Figure 1(i), it was a nonenveloped viral particle with a diameter of approximately (30–35) nm. All the previous results indicated that a PSV strain was successfully isolated in LLC-PK and ST cells, which was named as PSV HNHB-01 strain. We purified the PSV with the plaque assay at the PSV HNHB-01 P5, P10, and P20 on the ST cell. After the cell infected with PSV, large clear plaques were evident under an agar overlay medium on the cells (Figure 1(k)). The ST positive plaque-clone was further serially passaged on the ST cells.

Currently, the PSV HNHB-01 strain has been passaged over P100 on ST cells. The virus titers of PSV HNHB-01 P5, P30, P40, P60, and P100 were determined using TCID_50_ assay. The viral titers were 4.60, 9.30, 9.25, 9.67, and 10.50 lgTCID_50_/0.1 mL, respectively (Figure 1(l)).

### 3.2. Virus Growth Kinetics of PSV HNHB-01 Strain on ST Cells

**Figure 2:**
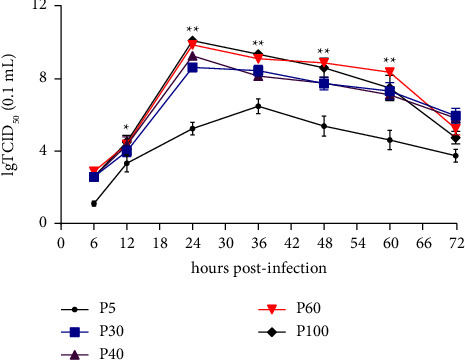
The growth kinetics of PSV HNHB-01 strain at selected passages in ST cells. ST cells were infected with HNHB-01 P5, P30, P40, P60, and P100 at MOIs of 0.01. The cells and supernatants were harvested together at the designated times and titrated with TCID_50_ infectivity assays. Asterisk (^*∗*^) indicates a significant difference between HNHB-01 P5 and HNHB-01 P100 (^*∗*^*P* < 0.05; ^*∗∗*^*P* < 0.01).

To analyze the biological characteristics of PSV HNHB-01 strain *in vitro*, the one-step growth curves of PSV HNHB-01 P5, P30, P40, P60, and P100 strains were all determined. Briefly, the ST cells were inoculated with HNHB-01 P5, P30, P40, P60, and P100 at MOIs of 0.01. Then, the cells and supernatants were harvested together at 6, 12, 24, 36, 48, 60, and 72 hpi, and virus titers were tested by TCID_50_ assays. The results showed that the virus titer of PSV HNHB-P5 peaked at 36 hpi (6.5 lgTCID_50_/0.1 mL) and then it began to decrease, whereas the virus titers of PSV HNHB-01 P30, P40, P60, and P100 reached the maximum titers of 8.78, 9.25, 9.78, and 10 lgTCID_50_/0.1 mL at 24 hpi, respectively (Figure 2). In conclusion, these results indicated that the adaptability of PSV HNHB-01 strain in ST cells gradually increased during its serial passage *in vitro* and has adapted to grow efficiently on ST cells around 30 passages.

### 3.3. Complete Genome Sequence and Phylogenetic Analysis of the Cell Culture-Adapted PSV HNHB-01 Strain

The complete genomes of PSV HNHB-01 P5, P30, P60, and P100 of ST cell culture-adapted PSV strains were sequenced and deposited in the GenBank database (the GenBank database numbers were ON375365, ON375366, ON375367, and ON375368, respectively). The length of the complete genomes excluding the 3′ ploy (A) tails were all 7,565 nt, including a 496 nt 5′- UTR sequence, a 6,989 nt polyprotein gene (L-VP4-VP2-VP3-VP1-2A-2B-2C-3A -3B-3C-3D) and a 80 nt 3′-UTR sequence. During the passage of the PSV HNHB-01 on ST cells, there was 9 nt changes, and resulted to 7 Aa changes. One Aa change (Ser changed to Asn at position 2020 nt of the HNHB-01 P5) was occured in the PSV HNHB-01 P30 strain when compared with the PSV HNHB-01 P5 strain. There existed three Aa changes (Tyr changed to Cys at position 544 nt, Leu changed to Ser at position 1,166 nt, Leu changed to Phe at position 1404 nt) on the PSV HNHB-01 P60 strain compared with the PSV HNHB-01 P30. There were three Aa changes (Cys changed to Tyr at position 544 nt, Tyr changed to His at position 1,238 nt, Ser changed to Pro at position 1,453 nt) on the PSV HNHB-01 P100 strain when compared with the PSV HNHB-01 P60. When the PSV HNHB-01 was serially passaged up to 100 times, there were 7 Aa changes in the complete genome on PSV HNHB-01 P100 strain when compared with the PSV HNHB-01 P5 strain.

**Figure 3:**
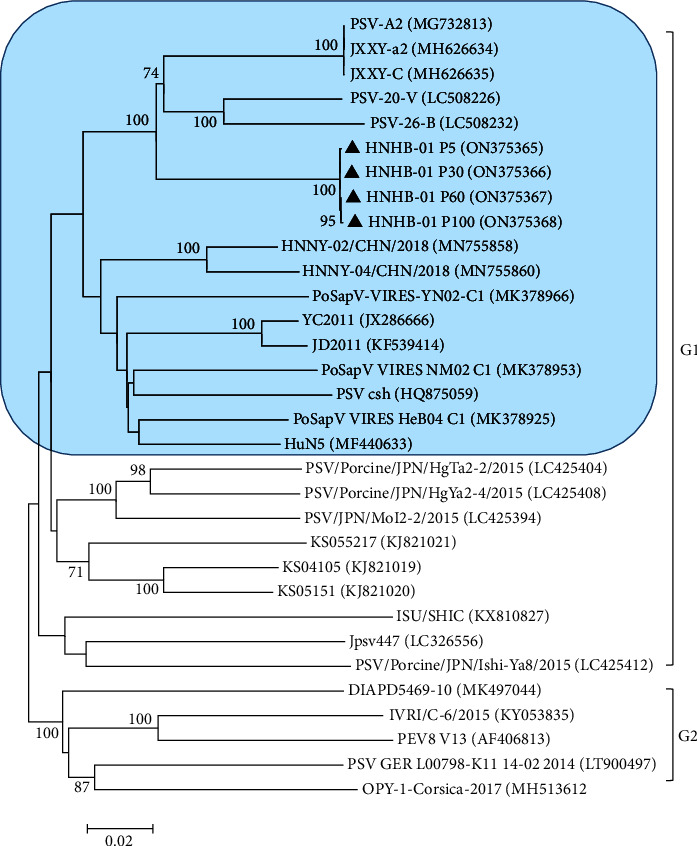
Phylogenetic analysis based on the complete sequences from different PSV strains. The phylogenetic tree was constructed from the aligned nucleotide sequences using the neighbour-joining method with MEGA 6.06 software (http://www.megasoftware.net) based on the whole genomes from PSV HNHB-01 strains (P5, P30, P60, and P100) and the other PSV strains obtained from NCBI. Bootstrap values were calculated with 1,000 replicates. Reference sequences obtained from GenBank are indicated by strain names and GenBank accession numbers. The black triangles indicate the PSV strains that identified by our laboratory. The China PSVs in this study were all in blue boxes.

Phylogenetic analyses were carried out using the nucleotide sequences of the full-length genomes of the PSV HNHB-01 strains (P5, P30, P60, and P100) and the other 28 PSV strains obtained from NCBI. It indicated that these PSV strains were classified into two distinct clusters (G1 and G2). The G1 cluster included the China, Japan, Zambia, and South Korean PSV strains and the cell culture-adapted HNHB-01 P5, P30, P60, and P100. The G2 cluster included the France, India, Italy, and Germany PSV strains (Figure 3). The PSV HNHB-01 P5, P30, P60, and P100 shared 86.3–88% nucleotide identities with the Japan PSV strains, 89.7–90.2% nucleotide identities with the Zambia PSV strains, and 84.5–86.8% nucleotide identities with the Germany and Korea PSV strains, and 87.5–90.4% nucleotide identity with the other Chinese PSV strains which were available in GenBank. The complete genomes of PSV HNHB-01 P5, P30, P60, and P100 shared approximately 99.9–100% nucleotide identities.

### 3.4. Clinical Manifestations and Necropsy of Piglets Challenged with PSV HNHB-01

**Figure 4:**
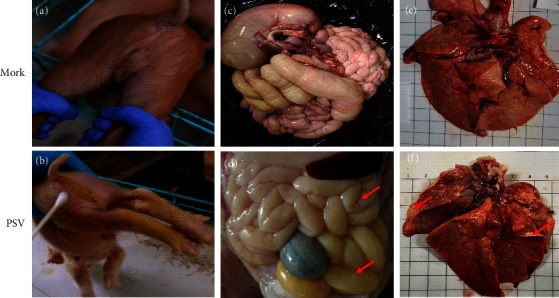
Clinical assessment of piglets challenged with PSV strain HNHB-01. (a and c) Five-day-old piglets infected with DMEM medium. (b and d) 5-day-old piglets infected with PSV. (e) The lung of 5-day-old piglets infected with DMEM medium. (f) The lung of 5-day-old piglets infected with PSV.

To evaluate the pathogenicity of PSV strain HNHB-01, 5-day-old piglets were orally challenged with PSV HNHB-01 P30 (10^9^ TCID_50_ per pig). All the piglets from the control group were active and eating well, and had no obvious clinical symptoms (Figure 4(a)). The piglets infected with PSV HNHB-01 showed watery diarrhea, twitching, and anorexia at 2 dpi (Figure 4(b)). When necropsied at 3 dpi, the PSV-inoculated piglets showed obvious intestine lesions characterized by dilatation and accumulation of yellow fluids in the intestinal lumen (Figure 4(d)), and the consolidation was found in the lungs (Figure 4(f)). No significant lesions were found in the uninfected piglets (Figures 4(c) and 4(e)).

### 3.5. Virus Distribution in the PSV Strain HNHB-01-Infected Piglets

**Figure 5:**
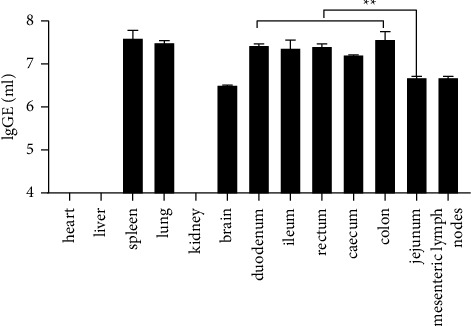
Viral distributions in various tissues of the PSV infected-piglets. The virus copies (lgGE/mL of total RNA) were the mean virus copy numbers of three piglets. Virus distribution was detected using qRT-PCR. Asterisk (^*∗*^) indicates a significant difference between duodenum, ileum, colon, rectum, and caecum with jejunum (^*∗*^*P* < 0.05; ^*∗∗*^*P* < 0.01).

To explore the PSV distribution in the organs of the infected piglets, viral load in different tissue samples were conducted by qRT-PCR. PSV viral RNAs could be detected in brain, lung, spleen, mesenteric lymph nodes, duodenum, jejunum, ileum, colon, caecum, and rectum, while the PSV viral RNAs were not detected in heart, liver, and kidney. Moreover, the levels of viral RNAs were higher in the duodenum, ileum, colon, rectum, and caecum than in the jejunum (< 0.01) (Figure 5). All the corresponding tissues from the control group were negative for PSV.

### 3.6. Histopathology and Immunohistochemistry on the Tissue Lesions of the PSV-Infected Piglets

**Figure 6:**
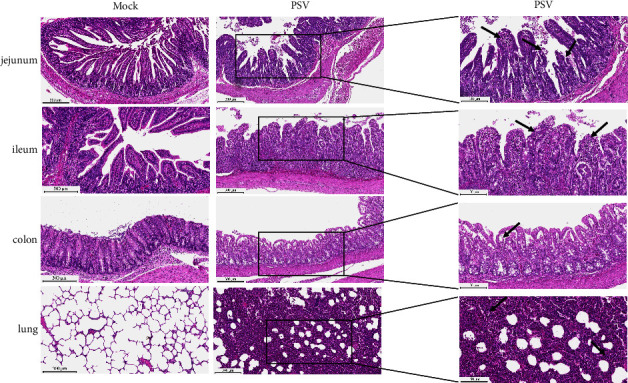
Histology of tissue sections of the piglets inoculated with PSV HNHB-01-P30. The intestinal tissues (jejunum, ileum, and colon) and lung from the 5-day-old piglets inoculated with PSV HNHB-01 were stained via the H&E. The arrows indicate the typical histological lesions in the detected tissues.

**Figure 7:**
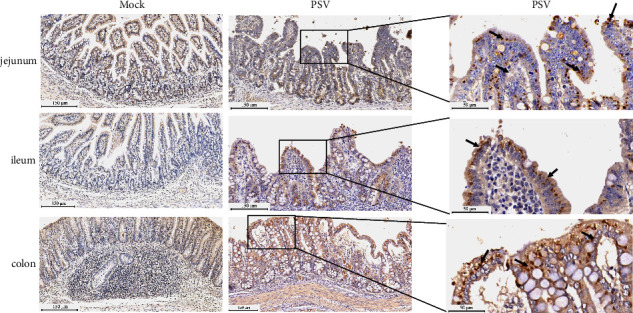
Immunohistochemical analysis of the intestine tissues from piglets inoculated with PSV HNHB-01. The jejunum, ileum, and colon from tissues were collected at 3 dpi, and the PSV antigens were detected with the PSV specific monoclonal antibody. The PSV VP1 antigens were indicated by the arrows in the detected tissues.

The diseased tissues were also examined by histopathology and immunohistochemistry. The histopathological examination of the piglets infected with HNHB-01 showed intestinal necrosis, severe intestinal villi shedding and atrophy, intestinal villous epithelium swells with numerous vacuoles and bleeding between connective tissues in the jejunum and ileum. The lesions in the colon were characterized by slight damage of the mucosa, villi shedding, bleeding, and inflammatory cell infiltrate. The alveolar septa in the lung were thickened, and filled with numerous erythrocytes (Figure 6). Consistent with the histopathological results, PSV antigens could be detected in jejunum, rectum, and colon, but could not be detected in lung from the PSV-infected piglets. No lesions were found in the intestines and lungs of the uninfected piglets (Figure 7).

### 3.7. Epidemiological Investigation of PSV Infections in Swine Herds in Henan Province, China

**Figure 8:**
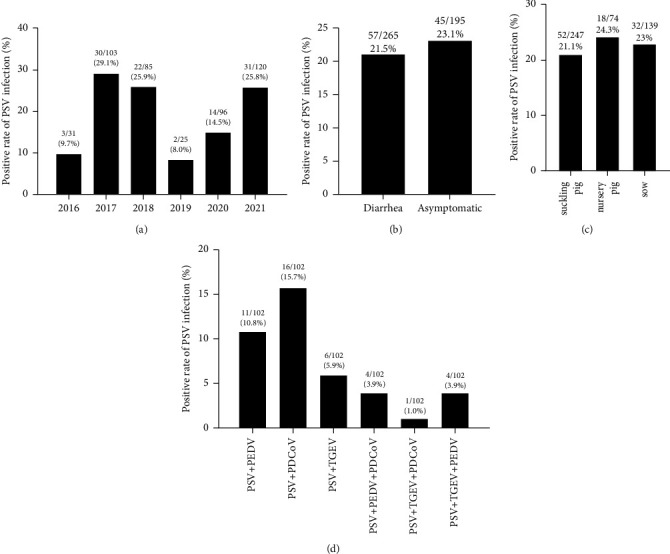
Detection of PSV infections in pigs in Henan Province, China. (a) The positive rates of PSV in different years. (b) The positive rates of PSV infection in diarrhea pigs and asymptomatic pigs. (c) The positive rates of PSV in the different growing stage. (d) The positive rate of PSV coinfection with PEDV, PDCoV, and TGEV in 102 PSV positive samples.

To evaluate the frequency of PSV infection in swine herds in Henan Province, China, 460 clinical samples were collected. As shown in Figure 8(a), 102 PSV-positive samples were detected from these samples and the total PSV-positive rate was 22.17% (102/460), and the positive rates of PSV in different years were further analyzed. PSV was identified in 9.7% (3/31) of the clinical samples collected in 2016, 29.1% (30/103) in 2017, 25.9% (22/85) in 2018, 8.0% (2/25) in 2019, 14.5% (14/96) in 2020, and 25.8% (31/120) in 2021. These results showed that the PSV has circulated in swine herds in recent years.

Among the 460 samples, 265 samples were from diarrhea pigs and 195 samples were collected from the asymptomatic pigs. There were 57 PSV-positive samples in the diarrhea samples (21.5%), and 45 PSV-positive samples in the asymptomatic samples (23.1%) (Figure 8(b)). Moreover, the positive proportion of PSV infection was 21.1% (52/247) in suckling piglets, 24.3% (18/74) in nursery pigs, and 23% (32/139) in sow pigs (Figure 8(c)). These data also suggested that PSV can be detected in diarrhea and asymptomatic pigs, and can infect pigs with multiple growth stages.

Our detection also found that the coinfections of PSV with PEDV, PDCoV, and TGEV were common in pigs. 11 out of 102 PSV positive samples were proved to be PEDV coinfection, with the positive rate of 10.80%. In addition, TGEV was identified in 6 out of 102 (5.9%) PSV positive samples and the coinfection of PSV with PDCoV was 15.7% (16/102) (Figure 8(d)).

### 3.8. Complete Genome Sequence and Phylogenetic Analysis of the Representative PSV Epidemic Strains

The whole genome sequences of eight representative PSV strains were determined and named as HNNY-01/CHN/2018, HNNY-02/CHN/2018, HNNY-03/CHN/2018, HNNY-04/CHN/2018, HNHB-01/CHN/2016, HNHB-02/CHN/2016, HNXX-01/CHN/2017, and HNXX-02/CHN/2017, and their sequences were uploaded to Genbank database with the GenBank numbers of MN939541, MN939542, MN939543, MN939544, MN755057, MN755058, MN755059, and MN755060, respectively. The molecular characterization of the eight PSV strains was further analyzed. The results showed that the complete genomic sequences of the eight strains were between 7,510 and 7,576 nucleotides in length, and these sequenced PSV strains had the same number of nucleotides in ORF regions, but had different nucleotides in the 5′-UTR and 3′-UTR. The nucleotide homology between the whole genome sequences of the PSV strains sequenced in this study ranged from 88% to 99.9% and from 82.0% to 90.1% with the 37 reference PSV strains obtained from NCBI.

**Figure 9:**
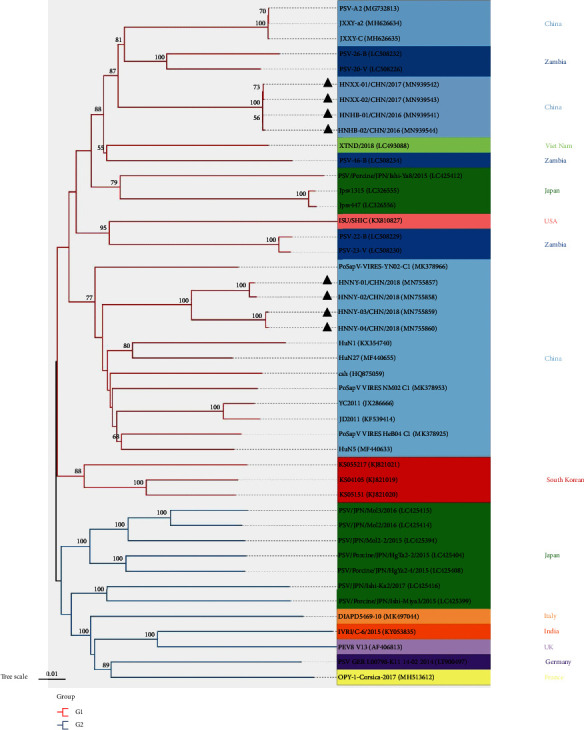
Phylogenetic tree constructed from the complete genome sequences of PSV strains. The eight strains sequenced in this study are indicated by black triangles.

The phylogenetic analysis based on whole genome sequences indicated that the PSV strains were divided into two groups, Group 1 (G1) and Group 2 (G2). The G1 cluster included the China, Vietnamese, Zambian, Japanese, Korean, USA, and the representative PSV epidemic strains obtained from this study, and the GII cluster included the European, Indian, and Japanese strains (isolated from 2015 to 2017). In addition, the PSV HNXX-01/CHN/2017, HNXX-02/CHN/2017, HNHB-01/CHN/2016, and HNHB-02/CHN/2016 strains determined in this study closely related to the Zambian PSV-26-B (LC508232) and PSV-20-V (LC508226) strains. While the PSV HNNY-01/CHN/2018, HNNY-02/CHN/2018, HNNY-03/CHN/2018, and HNNY-04/CHN/2018 were located together with the most China PSV strains, indicating that the origin of the eight PSV strains determined in this study may be different (Figure 9).

### 3.9. Recombination Analysis of PSV Strains Identified in This Study

**Figure 10:**
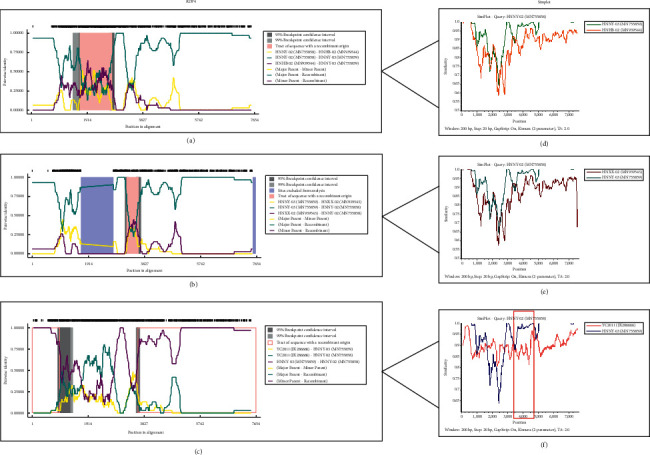
Recombination analysis of PSV strains using the RDP4 (left) and simplot (right). The red box is the possible sites of recombination.

In order to further analyze the association between these PSV strains and those of the existing isolates, the recombination analysis was performed with RDP4 and Simplot software. First, the eight strains sequenced in this study were compared using the RDP4 software and two recombination events were predicted (Figures 10(a) and 10(b)). In the first predicted recombination event, the recombination events detected in the HNNY-03/CHN/2018 strain, when the HNNY-02/CHN/2018 was used as the major parent of the recombinant strain, and the HNHB-02/CHN/2016 was used as the minor parent of the recombinant strain (Figure 10(a)). The probability of the HNNY-03/CHN/2018 as a recombinant strain was 46% (Table 2). In the second recombination event, the recombination events were detected in HNNY-02/CHN/2018 strain and the predicted probability was 46.5% (Table 2); here, the HNNY-03/CHN/2018 strain was the major parent of the recombinant strain and HNXX-02/CHN/2017 strain was the minor parent of the recombinant virulent strain (Figure 10(b)). Then, the two recombination events were further verified using Simplot software. The results showed that there was no genetic recombination between the eight representative PSV strains (Figures 10(d) and 10(e)).

Furthermore, we compared the eight representative PSV strains with the 25 PSV reference genome sequences (within the G1 group in Figure 9). Notably, we detected the recombination signal in the HNNY-02/CHN/2018 genome sequence, with the predicted recombinant probability of 63.3% (Table 2), when the YC2011 strain was used as the major parent strain, and the HNNY-03/CHN/2018 strain was used as the minor parent strain (Figure 10(c)). Then, the recombination events were further verified using Simplot software, the results showed the presence of multiple crossover site, which could tentatively confirm that the recombination events were occurred in our sequenced PSV HNNY-02/CHN/2018 strain, and the possible sites of recombination events was located at the nucleotide position 3,349 bp to 4,819 bp of PSV HNNY-02/CHN/2018, which is located the downstream of 2C region of PSV (Figure 10(f)).

## 4. Discussion

PSV can cause various swine diseases and threat the pig industry development. The PSV infection frequently goes unnoticed or overlooked in swine herds because of the subclinical manifestations and coinfections with other pathogens [28, 29]. In this study, we isolated the PSV HNHB-01 strain in ST and LLC-PK from a clinical PDCoV-positive intestinal content sample of a diarrheic pig, and the genetic diversity of PSV HNHB-01 strain during passage on ST cell were analyzed. The pathogenicity of PSV HNHB-01 strain on 5-day-old piglets was characterized. Moreover, the molecular epidemic investigation revealed that PSV was highly prevalent in pigs in Henan province, and was common in coinfection with PEDV, TGEV, and PDCoV. The information presented in this study is important for future research studies of the pathogenic mechanism, evolutionary characteristics, and the development of vaccines against PSV.

PSV could be detected and/or isolated from feces of healthy as well as diarrheal piglets [13, 15]. Notably, pigs that have recovered from PSV infection can still excrete the virus and which is an important source of infection. In this study, the high PSV prevalence was found in pigs in Henan province, with the positive rates of 22.17%. The PSV-positivity rate of 11.22% was reported in 4 farms in western Jiangxi Province from December 2018 to March 2019 [30], and the PSV was detected in Anhui, Jiangsu, and Guangxi [8]. These results suggested that the PSV has circulated in many swine herds in China, and overall PSV infection in different pig populations in China should be conducted. Moreover, our study revealed that the prevalence of PSV in asymptomatic and diarrheic pigs was 23.1% and 21.5%, respectively. These results indicated that PSV transmission in healthy pigs was the risk infection factor for the piglets, so continuous surveillance studies will be important to monitor the PSV prevalence in apparently asymptomatic pigs. Furthermore, the coinfections of PSV with PEDV, PDCoV, PTV, and PEV in natural infections were common in previous reports [9, 29]. The positive rate of PSV and PEDV coinfection was 20.29% in diarrhea samples [8]. Our detection also found that coinfections of PSV with PDCoV, TGEV, and PEDV were common in diarrheal pigs, and the positive rate of PSV and PDCoV coinfection was 15.7%, indicating that PSV may contribute collectively to diarrhea of pigs along with other porcine pathogens. Therefore, PSV cannot be ignored when controlling the diarrhea of pigs.

In the current study, a PSV strain happened to be isolated in ST and LLC-PK cells from the intestinal contents of a clinical PDCoV-infected pig. We have attempted to isolate PDCoV from this swine intestinal content sample, and the obvious visible CPEs were observed, but it was negative for PDCoV by viral RNA and antigen detection. Finally, the cell culture was tested by next generation sequencing, and was identified as PSV. PSV was capable of infecting several cell lines, including ST, porcine kidney (PK-15), IPEC-J2, 293T, and BHK-21 cells [8, 15]. In this study, we used the ST and LLC-PK cells to isolate and serially passage the PSV HNHB-01 strain. Our results identified that the ST and LLC-PK cells are all susceptible to PSV infection. The PSV HNHB-01 strain has been successfully serially propagated in cell culture for over 100 passages. The biological characteristics in the HNHB-01 at different passages, including P5, P30, P40, P60, and P100, showed that PSV HNHB-01 obtained susceptibility and adaptability to ST cells gradually as it was serially passaged.

The pathogenicity of PSV HNHB-01 was evaluated in 5-day-old piglets. After the piglets infected with PSV HNHB-01 for 48 h, diarrhea, respiratory distress occurred consecutively in the virus-infected piglets. These symptoms were similar to the clinical symptoms caused by the PSV strains SHCM2019 and PSV-JXXY-a2 [8, 31], revealing that the PSV HNHB-01 strain was pathogenic in piglets, and can replicate efficiently in the intestine and induce diarrhea in piglets. Previous study showed that PSV can be detected in duodenum, jejunum, ileum, caecum, colon, rectum, lung, brain, and mesenteric lymph nodes [8, 32], which was consistent with our results. Histopathologic changes were mainly occurred in the intestines and lungs. Immunohistochemical analysis showed that a large number of PSV antigens were detected in the jejunums, ileums, and colon of the PSV-infected piglets. These results were also similar to those described in the previous reports involving pigs infected with PSV.csh strain [6]. All these results indicated that the PSV HNHB-01 is a pathogenic strain.

The genetic changes in the complete genomes of PSV HNHB-01 during serial passages in cell cultures were determined and analyzed. The PSV HNHB-01 was relatively stable during the 100 passages in cell culture, with only 9 nt changes (result to 7 Aa changes). While the influence of these nt or Aa mutants on PSV replication and pathogenicity needs further investigation. Simultaneously, the complete genomes of the eight representative PSV epidemic strains were sequenced and analyzed in the current study. Homology analysis showed that the nucleotide homology between the whole genome sequences of PSV sequenced in this study ranged from 88% to 99.9% and the eight representative PSV strains shared 82%–90.1% nucleotide identity with the reference PSV strains. Phylogenetic analysis demonstrated that these PSV strains are classified into two distinct clusters. However, the eight representative PSV strains were more closely related to PSVs previously detected in China than PSVs isolated from other countries, suggesting that the eight PSV strains are all Chinese pandemic strain and our isolated PSV HNHB-01 strain is a potential vaccine candidate.

In summary, we isolated and identified a prevalent strain PSV HNHB-01 that found on the PDCoV coinfected pigs. This virus was relatively stable during serially passaged in cell culture, with only 9 nt changes and 7 Aa changes within the 100 passages. The PSV HNHB-01 strain was highly enteropathogenic in 5-day-old piglets. The result of molecular epidemiology investigation indicated that PSV was highly prevalent in swine herd in Henan province, China and can be coinfected with a variety of diarrhea coronaviruses. Collectively, our study provided basic information for the investigation of PSV pathogenesis, and also for the development of potential vaccines against PSV.

## Figures and Tables

**Table 1:** The primer information for complete genome of PSV.

Primers	Sequences (5′-3′)	Primer position	Product (bp)
PSV-F1	GCGTTGAAATGGGTGTGGGGTA	1–19	2,977
PSV-R1	CGGGTTGATACCATAAGAGGCA	2,955–2,977	

PSV-F2	CAGTAGAACCTCCCTTGCTATG	634–656	3,963
PSV-R2	GTTCCAGGAGCACCATGTATAA	4,575–4,597	

PSV-F3	TCTGTGGAGCCGGTTTAATATC	3,684–3,706	2,452
PSV-R3	CAGGTCTTGATGGTGTGTATCT	6,113–6,135	

PSV-F4	CCAGTTGAGGCAGAGGTTATTC	5,342–5,364	2,214
PSV-R4	TAGGCGGCTTAGGGTCTATT	7,536–7,556	

PSV-F5	CCTTCCTGTGTCATCCTGTATTT	7,254–7,277	312
Oligo-dT	TTTTTTTTTTTT		

**Table 2:** Recombination events identified by RDP4 and simplot software.

Recombinants	Major parent	Minor parent	*Breakpoint positions* ^*a*^ *(bp)*		Recombinant scores^b^ (%)	Recombinant positions^c^ (bp)
			Beginning	End		
HNNY-03/CHN/2018	HNNY-02/CHN/2018	HNHB-02/CHN/2016	1,662	2,767	46	—
HNNY-02/CHN/2018	HNNY03/CHN/2018	HNXX02/CHN/2017	3,224	3,632	46.5	—
HNNY-02/CHN/2018	YC2011	HNNY-03/CHN/2018	3,614	952	63.3	970–4,819

Recombinant region breakpoints at nucleotide positions of predicted recombinant strains. ^a^score greater than 40 and less than 60 means that the strain is likely to be a recombinant strain; ^a^score less than 40 means that the strain is likely not to be a recombinant strain. ^b^A score greater than 60 means that the strain is almost certainly a recombinant strain; ^c^Reorganization breakpoints determined after Simplot software analysis. —Reorganization breakpoint not detected.

## Data Availability

Data are available upon request (huhui2001@163.com).

## References

[1] Tseng C., Tsai H. (2007). Sequence analysis of a duck picornavirus isolate indicates that it together with porcine enterovirus type 8 and simian picornavirus type 2 should be assigned to a new picornavirus genus. *Virus Research*.

[2] Son K., Kim F. D., Kwon J. (2014). Full-length genomic analysis of Korean porcine Sapelovirus strains. *PLoS One*.

[3] Racaniello R. V. (2013). Picornaviridae: the viruses and their replication. *fields Virology*.

[4] Schock A., Gurrala R., Fuller H. (2014). Investigation into an outbreak of encephalomyelitis caused by a neuroinvasive porcine sapelovirus in the United Kingdom. *Veterinary Microbiology*.

[5] Chen Q., Zheng Y., Guo B. (2016). Complete genome sequence of porcine sapelovirus strain USA/IA33375/2015 identified in the United States. *Genome Announcements*.

[6] Lan D., Ji W., Yang S. (2011). Isolation and characterization of the first Chinese porcine sapelovirus strain. *Archives of Virology*.

[7] Oberste M., Maher K., Pallansch M. (2002). Molecular phylogeny and proposed classification of the simian picornaviruses. *Journal of Virology*.

[8] Li Y., Du L., Jin T. (2019). Characterization and epidemiological survey of porcine sapelovirus in China. *Veterinary Microbiology*.

[9] Kim D. S., Kang M. I., Son K. Y. (2016). Pathogenesis of Korean Sapelovirus A in piglets and chicks. *Journal of General Virology*.

[10] Ibrahim Y., Zhang W., Werid G. (2022). Isolation, characterization, and molecular detection of porcine sapelovirus. *Viruses*.

[11] Donin D. G., de Arruda Leme R., Alfieri A. F., Alberton G. C., Alfieri A. A. (2014). First report of Porcine teschovirus (PTV), Porcine sapelovirus (PSV) and Enterovirus G (EV-G) in pig herds of Brazil. *Tropical Animal Health and Production*.

[12] Boros Á., László Z., Pankovics P. (2020). High prevalence, genetic diversity and a potentially novel genotype of Sapelovirus A (Picornaviridae) in enteric and respiratory samples in Hungarian swine farms. *Journal of General Virology*.

[13] Ray P., Desingu P., Kumari S. (2018). Porcine sapelovirus among diarrhoeic piglets in India. *Transboundary and emerging diseases*.

[14] Vilar M., Peralta B., García-Bocanegra I. (2016). Distribution and genetic characterization of Enterovirus G and Sapelovirus A in six Spanish swine herds. *Virus Research*.

[15] Bai H., Liu J., Fang L. (2018). Characterization of porcine sapelovirus isolated from Japanese swine with PLC/PRF/5 cells. *Transboundary and emerging diseases*.

[16] Knowles N., Buckley L., Pereira H. (1979). Classification of porcine enteroviruses by antigenic analysis and cytopathic effects in tissue culture: description of 3 new serotypes. *Archives of Virology*.

[17] Zhao T., Cui L., Yu X., Zhang Z., Shen X., Hua X. (2019). Entry of sapelovirus into IPEC-J2 cells is dependent on caveolae-mediated endocytosis. *Virology Journal*.

[18] Zhang H., Liang Q., Li B. (2019). Prevalence, phylogenetic and evolutionary analysis of porcine deltacoronavirus in Henan province, China. *Preventive Veterinary Medicine*.

[19] Liang Q., Zhang H., Li B. (2019). Susceptibility of chickens to porcine deltacoronavirus infection. *Viruses*.

[20] Dong N., Fang L., Yang H. (2016). Isolation, genomic characterization, and pathogenicity of a Chinese porcine deltacoronavirus strain CHN-HN-2014. *Veterinary Microbiology*.

[21] Hu H., Jung K., Vlasova A. (2015). Isolation and characterization of porcine deltacoronavirus from pigs with diarrhea in the United States. *Journal of Clinical Microbiology*.

[22] Jin X., Zhang Y., Yuan Y., Han L., Zhang G., Hu H. (2021). Isolation, characterization and transcriptome analysis of porcine deltacoronavirus strain HNZK-02 from Henan Province, China. *Molecular Immunology*.

[23] Li B., Zheng L., Li H., Ding Q., Wang Y., Wei Z. (2019). Porcine deltacoronavirus causes diarrhea in various ages of field-infected pigs in China. *Bioscience Reports*.

[24] Zhang H., Han F., Shu X. (2021). Co-infection of porcine epidemic diarrhoea virus and porcine deltacoronavirus enhances the disease severity in piglets. *Transboundary and emerging diseases*.

[25] Martin D., Murrell B., Golden M., Khoosal A., Muhire B. (2015). RDP4: detection and analysis of recombination patterns in virus genomes. *Virus evolution*.

[26] Lole K., Bollinger R., Paranjape R. (1999). Full-length human immunodeficiency virus type 1 genomes from subtype C-infected seroconverters in India, with evidence of intersubtype recombination. *Journal of Virology*.

[27] Hao C., Ren H., Wu X. (2022). Preparation of monoclonal antibody and identification of two novel B cell epitopes to VP1 protein of porcine sapelovirus. *Veterinary Microbiology*.

[28] Donin D. G., Leme R. A., Alfieri A. F., Alberton G. C., Alfieri A. A. (2015). Molecular survey of porcine teschovirus, porcine sapelovirus, and enterovirus G in captive wild boars (Sus scrofa scrofa) of Parana state, Brazil. *Pesquisa Veterinária Brasileira*.

[29] Prodělalová J. (2012). The survey of porcine teschoviruses, sapeloviruses and enteroviruses B infecting domestic pigs and wild boars in the Czech Republic between 2005 and 2011. *Infection, Genetics and Evolution*.

[30] Yang T., Zhang L., Lu Y., Guo M., Zhang Z., Lin A. (2021). Characterization of porcine sapelovirus prevalent in western Jiangxi, China. *BMC Veterinary Research*.

[31] Li N., Tao J., Li B. (2021). Molecular characterization of a porcine sapelovirus strain isolated in China. *Archives of Virology*.

[32] Yang T., Yu X., Yan M. (2017). Molecular characterization of porcine sapelovirus in hunan, China. *Journal of General Virology*.

